# PGC-1α Signaling Increases GABA(A) Receptor Subunit α2 Expression, GABAergic Neurotransmission and Anxiety-Like Behavior in Mice

**DOI:** 10.3389/fnmol.2021.588230

**Published:** 2021-02-01

**Authors:** Taavi Vanaveski, Svetlana Molchanova, Dan Duc Pham, Annika Schäfer, Ceren Pajanoja, Jane Narvik, Vignesh Srinivasan, Mari Urb, Maria Koivisto, Eero Vasar, Tönis Timmusk, Rimante Minkeviciene, Ove Eriksson, Maciej Lalowski, Tomi Taira, Laura Korhonen, Vootele Voikar, Dan Lindholm

**Affiliations:** ^1^Medicum, Department of Biochemistry and Developmental Biology, University of Helsinki, Helsinki, Finland; ^2^Minerva Foundation Institute for Medical Research, Helsinki, Finland; ^3^Institute of Biomedicine and Translational Medicine, University of Tartu, Tartu, Estonia; ^4^Quretec Ltd., Tartu, Estonia; ^5^Molecular and Integrative Biosciences Research Programme, Faculty of Biological and Environmental Sciences, University of Helsinki, Helsinki, Finland; ^6^Protobios LCC, Tallinn, Estonia; ^7^Department of Chemistry and Biotechnology, Tallinn University of Technology, Tallinn, Estonia; ^8^Meilahti Clinical Proteomics Core Facility, HiLIFE, University of Helsinki, Helsinki, Finland; ^9^Department of Biomedical Proteomics, Institute of Bioorganic Chemistry, Polish Academy of Sciences, Poznań, Poland; ^10^Department of Veterinary Biosciences, Faculty of Veterinary Medicine and Neuroscience Center, HiLIFE, University of Helsinki, Helsinki, Finland; ^11^Department of Child and Adolescent Psychiatry and Department of Biomedical and Clinical Sciences, Linköping University, Linköping, Sweden; ^12^Neuroscience Center and Laboratory Animal Center, HiLIFE, University of Helsinki, Helsinki, Finland

**Keywords:** GABA-A receptors, PGC-1α, PPARγ, pioglitazone, gene expression, neurotransmission, anxiety, behavioral test

## Abstract

Peroxisome proliferator-activated receptor-γ coactivator-1α (PGC-1α) is a master regulator of mitochondria biogenesis and cell stress playing a role in metabolic and degenerative diseases. In the brain PGC-1α expression has been localized mainly to GABAergic interneurons but its overall role is not fully understood. We observed here that the protein levels of γ-aminobutyric acid (GABA) type A receptor-α2 subunit (GABARα2) were increased in hippocampus and brain cortex in transgenic (Tg) mice overexpressing PGC-1α in neurons. Along with this, GABARα2 expression was enhanced in the hippocampus of the PGC-1α Tg mice, as shown by quantitative PCR. Double immunostaining revealed that GABARα2 co-localized with the synaptic protein gephyrin in higher amounts in the striatum radiatum layer of the hippocampal CA1 region in the Tg compared with Wt mice. Electrophysiology revealed that the frequency of spontaneous and miniature inhibitory postsynaptic currents (mIPSCs) was increased in the CA1 region in the Tg mice, indicative of an augmented GABAergic transmission. Behavioral tests revealed an increase for anxiety-like behavior in the PGC-1α Tg mice compared with controls. To study whether drugs acting on PPARγ can affect GABARα2, we employed pioglitazone that elevated GABARα2 expression in primary cultured neurons. Similar results were obtained using the specific PPARγ agonist, N-(2-benzoylphenyl)-O-[2-(methyl-2-pyridinylamino) ethyl]-L-tyrosine hydrate (GW1929). These results demonstrate that PGC-1α regulates GABARα2 subunits and GABAergic neurotransmission in the hippocampus with behavioral consequences. This indicates further that drugs like pioglitazone, widely used in the treatment of type 2 diabetes, can influence GABARα2 expression via the PPARγ/PGC-1α system.

## Introduction

Peroxisome proliferator-activated receptor-γ coactivator-1α (PGC-1α) is an important regulator of mitochondrial biogenesis, metabolism, and cell stress (Puigserver and Spiegelman, 2003; St-Pierre et al., 2006; Scarpulla, 2011). PGC-1α interacts with nuclear receptors, such as the peroxisome proliferator-activated receptor γ (PPARγ), affecting the expression of metabolic and other genes (Handschin and Spiegelman, 2006). PGC-1α itself is regulated by various factors in a cell and tissue-specific manner (Fernandez-Marcos and Auwerx, 2011; Lindholm et al., 2012; Martinez-Redondo et al., 2015). PPARγ is present in the nervous system, and stimulation of the receptor exerts neuroprotective effects by reducing inflammation and oxidative stress in the brain (Austin and St-Pierre, 2012; Chen et al., 2012; Zolezzi et al., 2013). It has further been shown that thiazolidinedione drugs acting via PPARγ, such as pioglitazone and rosiglitazone, can modulate behavior in experimental animals (Stopponi et al., 2011, 2013; de Guglielmo et al., 2017). However, the underlying mechanisms of how PPARγ signaling influences behavior and neuronal connectivity are not fully understood.

Previous studies demonstrated that PGC-1α is particularly expressed in interneurons in the brain, including those positive for the calcium-binding protein, parvalbumin (Cowell et al., 2007; Lucas et al., 2010). PGC-1α was further shown to regulate the expression of parvalbumin as well as other genes in the brain (Lucas et al., 2010, 2014), and mice lacking PGC-1α exhibited changes in inhibitory neurons and neurotransmission in brain cortex (Dougherty et al., 2014), striatum (McMeekin et al., 2018) and in cerebellum (Lucas et al., 2015). Recently, a role for PGC-1α in excitatory neurons of the hippocampus was reported (McMeekin et al., 2020), enlarging the functions of this coactivator to encompass several cell populations and neuronal circuits in the brain (Mortensen et al., 2012; Engin et al., 2018).

To investigate the role of PGC-1α in GABAergic transmission, we have here studied transgenic (Tg) mice overexpressing PGC-1α in neurons (Mudò et al., 2012; Mäkelä et al., 2016a). In the PGC-1α Tg mice, PGC-1α expression and protein levels are substantially increased in brain areas, such as hippocampus and frontal cortex, as compared with wild-type mice. Behavioral analyses performed revealed an increase in anxiety-like behavior in these mice compared with wild-types. Electrophysiological recordings showed that there was an increase in the frequency of spontaneous and miniature inhibitory postsynaptic currents (mIPSCs) in hippocampal neurons in PGC-1α Tg mice, indicative of an altered GABAergic neurotransmission. Using immunoblotting and qPCR we observed that the expression of GABARα2 subunit was enhanced in the hippocampus of Tg mice compared with Wt animals. These results demonstrate that stimulation of the PPARγ/PGC-1α system is involved in the regulation of GABARα2 expression and GABAergic neurotransmission in hippocampus with effects on behavior.

## Materials and Methods

### Animals Experiments

PGC-1α transgenic (Tg) mice with overexpression of PGC-1α under the Thy1 promoter in brain neurons were generated as previously described (Mudò et al., 2012). They were backcrossed over several generations with C57Bl6/J mice that also served as wild-type (Wt) controls (Mäkelä et al., 2016a). The PGC-1α Tg and wild-type mice were bred in the same room and kept under identical conditions during growth and for experiments. Experiments were approved by the ethics committees and carried out in accordance with the European Communities Council Directive (86/609/EEC). Every attempt was made to reduce sample size and to minimize pain and suffering of animals. The number of animals used is given under the different experimental settings below.

### Immunohistochemistry

Eight Wt and eight PGC-1α Tg male mice aged 4 months were deeply anesthetized with pentobarbital (120 mg/kg, i.p.) and transcardially perfused with PBS (pH 7.6) followed by 4% PFA in 1× PBS (Sokka et al., 2007). Whole brains were removed from the skull and fixed in 4% PFA for 24 h, immersed in 30% sucrose for 3 days and cryofrozen in isopentane. 20 μm thick sections from a region 1.5–2.1 mm posterior to Bregma were cut in the coronal plane on a cryostat (CM3050S, Leica Biosystems, Wetzlar, Germany). Sections were washed for 5 min in PBS, treated with 0.3% hydrogen peroxidase in PBS for 20 min, washed in PBS, and incubated for 1 h min with normal horse serum (Vector Lab) and 0.01% Triton X-100 in PBS. Primary GABARα2 antibody (diluted 1:1000, sc-7350, Santa Cruz Biotechnology) was added overnight at 4°C, followed by secondary biotin-conjugated antibodies (1:700, no. 31820, Thermo Fisher) for 2 h at room temperature (RT). Sections were further incubated for 30 min at RT with ABC reagent (VECTASTAIN^®^ Elite^®^ ABC-Peroxidase Kit, PK-6100, Vector Laboratories). Diaminobenzidine (DAB) reaction was performed with DAB substrate (Peroxidase Substrate Kit, SK-4100, Vector Laboratories) and stopped using double-distilled water. Sections were then mounted onto slides, dehydrated, cleared in xylene, and mounted with DPX (Biolab). Images were generated using 3DHISTECH Panoramic 250 FLASH II digital slide scanner at Genome Biology Unit, HiLIFE and the Faculty of Medicine, University of Helsinki. Control samples were without primary antibodies.

### Double-Staining of GABARa2 and Gephyrin in CA1 Region of Hippocampus

Brain sections from five Wt and PGC-1α Tg male mice aged 4 months were prepared as above and treated with 0.01 M sodium citrate buffer with 0.05% Tween-20 (pH 6) for 20 min at +95°C for antigen retrieval. Sections were washed and tissue permeabilized in 1% BSA, 10% normal donkey serum and 0.2% Tween-20 in PBS for 1h at RT. Primary rabbit anti-GABARα2 (1:1,000, no. 224 103, Synaptic Systems) and mouse anti-Gephyrin antibodies (1:1,000, no. 147 011, Synaptic systems) were added and sections incubated for 20 h at +4°C, washed and Alexa Fluor 488-conjugated donkey anti-rabbit (1:700, no. A-21206, Invitrogen, Thermo Fisher Scientific Inc., Carlsbad, CA, United States) and Alexa Fluor 594-conjugated donkey anti-mouse (1:600, no. A-21203, Invitrogen, Thermo Fisher Scientific Inc., United States) secondary antibodies were added for 2 h at RT. Microscopic slides were treated as above.

The colocalization of GABARα2 and gephyrin was examined in the stratum radiatum (SR), stratum lacunosum-moleculare (SLM) in the CA1 and molecular layer in the dentate gyrus region of the hippocampus. Images were acquired from a single optical section using a Leica SP8 inverted confocal microscope (Leica, Germany) with a 63× glycerol-immersion objective (numerical aperture, 1.3). Image deconvolution was done with Huygens Professional. Colocalization of GABARa2 and gephyrin was assessed using the ImarisColoc tool of Imaris 9.3 (Bitplane, Oxford Instruments, Oxfordshire, United Kingdom). The intensity thresholds for both channels were automatically defined based on an algorithm developed earlier (Costes et al., 2004). We analyzed first the percentage of colocalized pixels out of total pixel number and then the individual channels to arrive at the percentage of gephyrin signals above threshold that colocalize with GABARα2 (Figure 3). Data are from five mice in each group, using the average of two hippocampal sections per animal with four images per section.

### Immunoblotting

Hippocampus and frontal cortex were dissected from seven Wt and seven PGC-1α Tg male mice aged 4 months and lysed using ice-cold RIPA buffer (150 mm NaCl, 1% Triton-X-100, 0.5% sodium deoxycholate, 1% sodium dodecyl sulfate (SDS), 50 mM Tris–HCl pH 7.4) supplemented with protease inhibitor cocktail (Roche) and phosphatase inhibitors (PhoStop; Roche) (Hyrskyluoto et al., 2014; Mäkelä et al., 2016a). Equal amounts of protein were subjected to SDS-PAGE and blotted onto nitrocellulose filters (Amersham Biosciences, Buckinghamshire, United Kingdom), which were incubated for 1 h in 5% skimmed milk or BSA, in TBS-T (50 mM Tris–HCl pH 7.5, 150 mM NaCl, 0.1% Tween 20), and then with primary antibodies overnight at 4°C. These included antibodies against GABARα2 (diluted 1:1,000; no. 822-GA2C, PhosphoSolutions, Aurora, CO, United States), GABARα1 (diluted 1:1,000; no. 812-GA1N, PhosphoSolutions, United States), PGC-1α (1:2000; Merck Millipore, Billerica, MA, United States), and ß-actin that was used as control (1:1,000; no 2066, Sigma). After washing the filter was incubated with horseradish peroxidase-conjugated secondary antibodies (1:2,500; Jackson ImmunoResearch Laboratories, West Grove, PA, United States), followed by detection using enhanced chemiluminescence (Thermo Fisher Scientific, Waltham, MA, United States). Quantifications were done using the ImageJ software and the levels of GABARs and PGC-1α were calculated using ß-actin as control.

### Quantitative PCR

Hippocampus and frontal cortex were dissected from five Wt and five PGC-1α Tg male mice aged 4 months, and RNA prepared using the RNeasy lipid tissue kit (Qiagen), followed by cDNA synthesis using Superscript VILO cDNA synthesis kit (Invitrogen). qPCR amplification was performed using the Light Cycler 480 II instrument (Roche Diagnostics, Basel, Switzerland) as described (Mäkelä et al., 2016a; Pham et al., 2019). Reactions were run in 96-well plate format in a final volume of 10 μl, and contained 2 μl cDNA and 100 μM forward (F) and reverse primers (R) in 1× SYBR Green Master Mix (Roche). The reaction was carried out at 95°C for 10 min followed by at 95°C for 15 s, 60°C for 20 s, and 72°C for 10 s using 40 cycles. Each sample was run in triplicates on a 96-well plate, and water was used as a negative control. Expression levels were calculated based upon the 2^–Δ^ ^Δ^ ^*CT*^ method using *Gapdh* as control. Primers are shown in Table 1.

**TABLE 1:** List of primer sequences.

Gene	Forward primer	Reverse primer
GABARα2	ACAAAAAGAGGATGGGCTTG	TCATGACGGAGCCTTTCTCT
GABARα1	TGAGCACACTGTCGGGAAGA	CAGCAGTCGGTCCAAAATTCT
GAPDH	GGGTTCCTATAAATACGGACTGC	CCATTTTGTCTACGGGACGA

### Neuronal Cultures

Neurons were prepared from brain of embryonic, E17-old rats and plated onto poly-ornithine (Sigma) coated six-well plates (Do et al., 2013; Mäkelä et al., 2016a; Srinivasan et al., 2020). Experiments had an ethical approval. Cells were cultured for up to 10 days in Neurobasal medium, and subsequently stimulated for 1–2 days with 1–10 μM pioglitazone or using the PPARγ agonist, N-(2-benzoylphenyl)-O-[2-(methyl-2-pyridinylamino) ethyl]-L-tyrosine hydrate (GW1929) (Mäkelä et al., 2016b). Immunoblotting was done essentially as described above employing cell lysates made using RIPA buffer consisting of 150 mM NaCl, 1 mM EDTA, 1% (v/v) NP-40, 0.25% (w/v) sodium deoxycholate, 50 mM Tris–HCl, pH 7.4, and 1% (w/v) SDS. 30 μg of protein was run on a SDS-polyacrylamide gel electrophoresis (SDS-PAGE), and proteins were transferred to nitrocellulose membrane (Amersham Biosciences, Buckinghamshire, United Kingdom). The membranes were blocked in 5% skimmed milk-Tris-buffered saline/0.1% (v/v) Tween 20 (TBS-T) for 1 h at RT, and primary antibody was added and incubated overnight at +4°C. These included anti-GABARα2 (diluted 1:1,000), anti-PGC-1α (1:2,000), and anti-ß-actin (1:1,000) that was used as control. After washing the filter was incubated with horseradish peroxidase-conjugated secondary antibodies (1:2,500; Jackson ImmunoResearch Laboratories, West Grove, PA, United States), followed by detection using enhanced chemiluminescence (Thermo Fischer Scientific, Waltham, MA, United States). Quantifications were done using the ImageJ software and the levels of GABARs and PGC-1α were calculated using ß-actin as control.

### Electrophysiology

Electrophysiological recordings were done essentially as described (Comhair et al., 2018). Four Wt and four PGC-1α TG male mice aged 2 months were deeply anesthetized with isoflurane, decapitated and the brain was quickly removed. Parasagittal hippocampal slices (300 μm-thick) were prepared using a vibratome (Leica VT1200S) in ice-cold dissection ACSF of following composition: 117 mM choline chloride, 2.5 mM KCl, 0.5 mM CaCl_2_, 1.25 mM NaH_2_PO_4_, 7 mM MgSO_4_, 26 mM NaHCO_3_, 15 mM glucose, and 95% O_2_/5% CO_2_. Slices were left to recover for 1 h at 34°C in recording ACSF with the MgSO_4_ concentration raised to 3 mM. After a recovery period, slices were transferred to the submerged recording chamber on the visually guided patch-clamp setup. Recordings were done at 30°C in ACSF of the following composition: 124 mM NaCl, 3 mM KCl, 1.25 mM NaH_2_PO_4_, 1 mM MgSO_4_, 26 mM NaHCO_3_, 2 mM CaCl_2_, 15 mM glucose, and 95% O2/5% CO_2_. Whole-cell voltage clamp recordings from CA1 pyramidal neurons were performed with glass microelectrodes (4–6 MΩ), filled with solution of the following composition: 120 mM CsCl, 0.022 mM CaCl_2_, 4 mM MgCl_2_, 10 mM HEPES, 0.1 mM EGTA, 5 mM QX-314, 4 mM MgATP, 0.5 mM Na_2_GTP. Membrane voltage was kept at −70 mV. Passive membrane properties (series resistance, input resistance, and capacitance) were monitored by injection of 5 mV voltage steps, and if the change in series resistance during the experiment exceeded 30%, the recording was discarded. Spontaneous inhibitory postsynaptic currents (sIPSCs) were recorded in the presence of AMPA and NMDA receptor inhibitors (20 μM CNQX and 5 μM L-689,560). For miniature inhibitory postsynaptic current (mIPSC) recordings, tetrodotoxin (TTX, 1 μM) was added to the solution. Data were acquired using a Multiclamp 700A amplifier (Axon Instruments, United States) and digitized at 20 kHz. Recordings were done with WinLTP 2.30 (WinLTP Ltd. and University of Bristol, United Kingdom) and analyzed in the Mini Analysis program 5.6.6 (Synaptosoft, United States). Automatic threshold search was used to detect synaptic events, and all events were verified manually. Amplitude, frequency and kinetics of events were calculated using build-in Mini Analysis algorithms.

### Behavioral Analyses

The mice were housed in an individually ventilated cage system (Mouse IVC Green Line; 391 mm × 199 mm × 160 mm; air inlet and outlet valves located in the cage lid, on top of the cage; the rate of air change was set at 75 times per hour with airspeed at the maximum level of 0.05 m/s; half of the cage covered by a wire bar food hopper; Tecniplast, Italy). Cage enrichment was provided by bedding (aspen chips, 4 HP, Tapvei, Estonia), nesting material (equal amounts of aspen strips, PM90L, Tapvei, Estonia and Sizzle Nest paper strands, Dates and Group, United Kingdom) and an aspen brick (100 mm × 20 mm × 20 mm, Tapvei, Estonia). Food (Global Diet 2916C, pellet 12 mm, Envigo, IN, United States) and water (filtered and UV-irradiated) were available *ad libitum*. Room temperature was 22 ± 2°C and relative humidity 50 ± 15%. The lights were on from 6:00 AM to 6:00 PM and experiments were carried out during this period. The cages were cleaned once per week, and animals were weighed before moving to a new cage. In total, 24 wild-types (Wt) and 20 PGC-1α transgenic (Tg) mice were examined using a battery of behavioral tests. Female (F) and male (M) mice were analyzed separately using four different groups: Wt F (*n* = 15), Wt M (*n* = 9), Tg F (*n* = 9), and Tg M (*n* = 11), and at the beginning of the experiments the mice were 169–170 days of age. Behavioral data was analyzed by two-way ANOVA or repeated measures (RM) two-way ANOVA, followed by Tukey’s unequal N HSD *post hoc* test as indicated under the respective tests.

#### Video Tracking

Mice were video-tracked by Noldus EthoVision XT 10 system (Noldus Information Technology, Wageningen, Netherlands). The distance traveled by the subjects and the time spent in pre-defined zones was recorded as well as defined behavior (vertical counts or rearings, grooming, head-dips).

#### Open Field

Mice were analyzed in an open field arena of 30 cm × 30 cm (Med Associates, St. Albans, VT, United States). The animal was released in the corner of the field; horizontal and vertical activity was recorded during the 30 min testing period by the software. The illumination in the open field was ∼150 lx. The peripheral zone was defined as a 6 cm wide corridor along the wall.

#### Elevated Plus-Maze

The maze consisted of two open arms (30 cm × 5 cm) and two closed arms (30 cm × 5 cm, inner diameter) connected by central platform (5 cm × 5 cm) and risen to 40 cm above the floor. The floor of each arm was light gray, and the closed arms had slightly transparent (15 cm high) side- and end-walls made of Plexiglas. The illumination level in all arms was ∼150 lx. The mouse was placed in the center of the maze facing one of the enclosed arms and observed for 5 min. The latency to the first open arm entry, number of open and closed arm entries (four paw criteria) and the time spent in different zones of the maze were measured. The number of head dips, rearings, and fecal boli was counted manually after the trial from video recordings.

#### Light–Dark Box

The test was carried out in the open field arena (30 cm × 30 cm, Med Associates, St. Albans, VT, United States) equipped with infrared light sensors detecting horizontal and vertical activity. The dark insert (non-transparent for visible light) was used to divide the arena into two halves, and an opening in the wall (a door with a width of 5.5 cm and height of 7 cm) allowed free movement from one compartment to another. Illumination in the center of the light compartment was ∼550 lx. The animal was placed in the dark compartment and allowed to explore the arena for 10 min. Distance traveled, number of rearing, latency to enter the light compartment, and time spent in different compartments were recorded.

#### T-Maze Spontaneous Alternation

The T-maze was made of gray PVC. Each arm measured 30 cm × 10 cm; a removable central partition extended from the center of the black goal wall of the T to 7 cm into the start arm. This modification prevented the mouse from seeing or smelling the non-chosen arm during the sample run, thus minimizing interfering stimuli. The entrance to each goal arm was fitted with a guillotine door. Each trial consisted of an information-gathering, sample run, followed immediately by a choice run. For the sample run, a mouse was placed in the start arm, facing away from the choice point with the central partition in place, the door on start arm closed and doors to goal arms opened. After opening the door on the start arm, the mouse was allowed to choose a goal arm and confined there for 10 s by lowering the respective guillotine door. Then, the central partition was removed, the mouse replaced to the start arm (door closed), the doors to both goal arms were opened again. This was followed by the opening of the start door, and the choice run was started. Alternation was defined as entering the opposite arm to that entered in the sample trial (whole body, including tail). Three trials were run per day with an inter-trial interval at least 1 h, on two consecutive days (six trials altogether).

### Statistical Analyses

The number of animals is given under the specific subheadings. For behavior experiments, the effect of gender was particularly considered, and statistical differences when noted were given separately. Values for expression data and immunoblots are given as means ± SEM of the averages in independent experiments. Unpaired Student’s *t*-test was used for imaging and electrophysiology data, and Student’s *t*-test or ANOVA followed by Dunnett *post hoc* test were used for qPCR and immunoblotting data (Graph Pad Prism version 4.0, La Jolla, CA, United States). *p* < 0.05 was considered as significant. Normality distribution of data was determined with the Shapiro–Wilks normality test (*p* < 0.01). Graphs with behaviors show values for each mouse and are presented as means with 95% confidence intervals. Behavioral data was analyzed by two-way ANOVA or RM two-way ANOVA, followed by Tukey’s unequal N HSD *post hoc* test where applicable (Statistica software, StatSoft, Tulsa, OK, United States).

## Results

### GABARα2 Subunits Are Increased in PGC-1α Overexpressing Transgenic Mice

**FIGURE 1:**
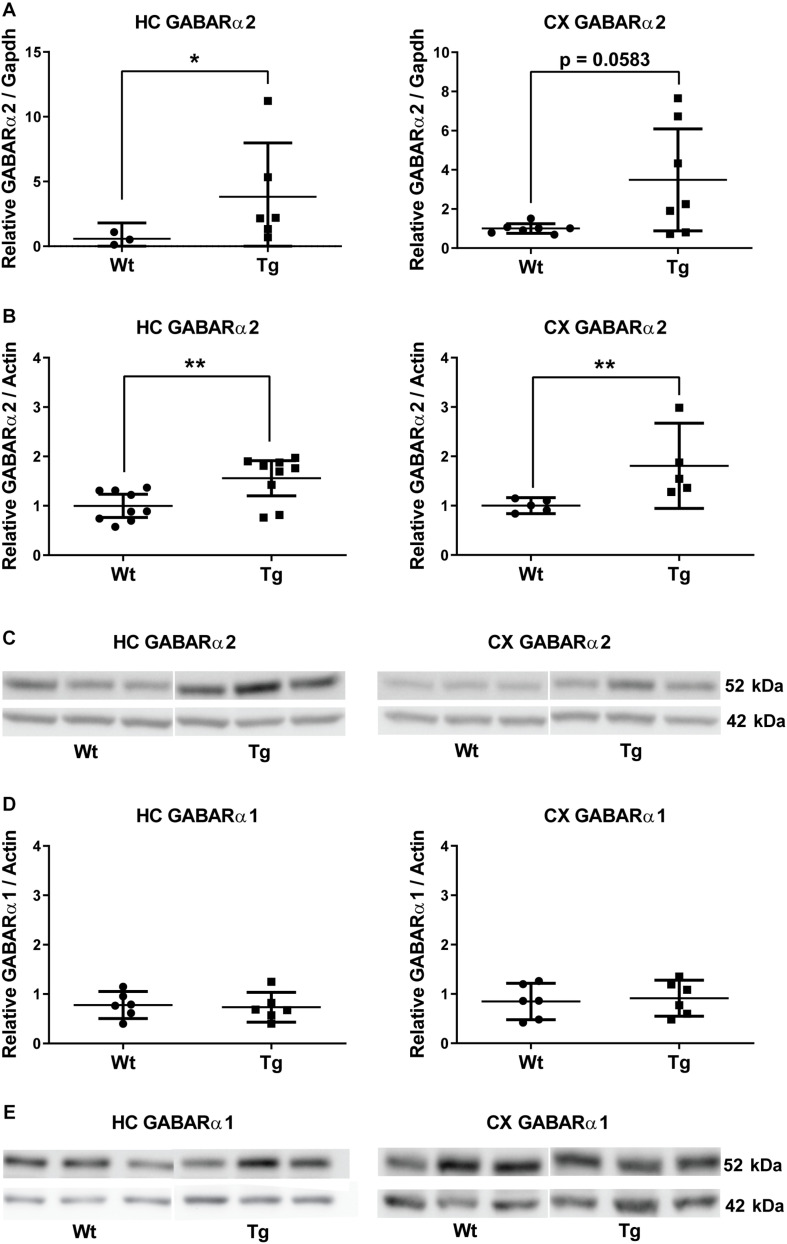
GABA-A receptor α2 subunit expression is increased in PGC-1α transgenic mice. **(A)** Quantitative PCR was performed as described in the section “Materials and Methods” using cDNA from the hippocampus (HC) and frontal cortex (CX) of 4-month old wild-type (Wt) and PGC-1α transgenic mice (Tg). Quantifications were done as described in the section “Materials and Methods” using *Gapdh* expression as control and Student’s *t*-test. Values are means ± SEM, *n* = 7. Left, HC; GABARα2 mRNA levels increased in the hippocampus of Tg mice compared with Wt animals. *p < 0.05. Right, CX. In the frontal cortex the difference was not significant between groups. *p* = 0.058. **(B)** Immunoblottings using lysates from the hippocampus (HC) and frontal cortex (CX) of 3-month old Wt and PGC-1α Tg mice were done using anti-GABARα2 antibody and anti-β-actin antibody as control. GABARα2 is shown at 52 kDa (upper lanes) and β-actin at 42 kDa (lower lanes). Quantification of GABARα2 levels was done using β-actin as control and Student’s *t*-test. Values are means ± SEM. *n* = 9 for HC, *n* = 6 for CX. **p < 0.01 for Tg vs. Wt. **(C)** Representative immunoblots showing GABARα2 at 52 kDa and β-actin as a control at 42 kDa. **(D)** Immunoblottings were done using anti-GABARα1 antibody and anti-β-actin antibody as control. Left, HC; Right, CX. GABARα1 levels were quantified using β-actin as control and Student’s *t*-test. Values are means ± SEM. *n* = 7 for HC, *n* = 6 for CX. There was no significant difference between groups. **(E)** Representative immunoblots showing GABARα1 at 52 kDa (upper lane) and β-actin as a control at 42 kDa (lower lanes).

We have previously shown that PGC-1α expression and protein levels are increased in brains of PGC-1α Tg mice in brain regions, including substantia nigra and hippocampus, compared with wild-type mice (Mudò et al., 2012; Mäkelä et al., 2016a). Furthermore, the PGC-1α Tg mice are protected against adverse effects of the neurotoxin, 6-hydroxydopamine, and against glutamate-mediated excitotoxicity in the hippocampus (Mudò et al., 2012; Mäkelä et al., 2016a). Proteomic analyses revealed alterations in several proteins associated with mitochondria and oxidative phosphorylation in the PGC-1α Tg mice (Mäkelä et al., 2016a). In these experiments, we observed an increase in the level of GABARα2 subunit prompting further investigations. qPCR analyses revealed that the expression of GABARα2 was increased in hippocampus of the PGC-1α Tg mice compared with Wt using *Gapdh* as control (Figure 1A). In frontal cortex, the variation between different animals was rather large in PGC-1α Tg mice and the change did not reach statistical significance (*p* = 0.058, *n* = 7) (Figure 1A). Immunoblotting experiments showed that the protein levels of GABARα2 were significantly increased in the hippocampus and frontal cortex of Tg mice compared with Wt and using ß-actin as control (Figures 1B,C). In contrast, the level of GABARα1 was not altered in the PGC-1α Tg mice as shown by immunoblotting (Figures 1D,E). Altogether, these results show that GABARα2 subunit level is increased in hippocampus and parts of the cortex in the Tg mice as compared with Wt.

**FIGURE 2:**
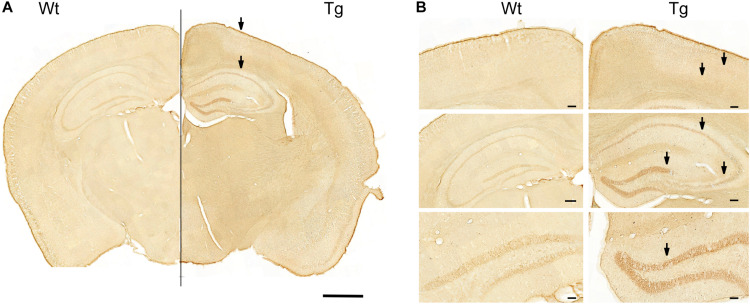
Immunostaining of GABA-A receptor α2 in PGC-1α Tg mouse brain. Immunostaining was done as described in the section “Materials and Methods” using anti-GABARα2 specific antibodies and brain sections from wild-type (Wt) and PGC-1α Tg mouse aged 4 months. **(A)** Combined brain. Coronal sections of Wt brain (Left) and Tg mouse (Right) were connected at the middle line. Hippocampus and parietal cortex are shown. Note higher intensity of staining in Tg mice compared with wildtype. Arrows indicate areas shown below. Size bar = 1 cm. **(B)** Higher magnifications. (Upper panels) GABARα2 immunopositive cells are present in layers of parietal cortex (arrows) and in ventral retrosplenial area at the middle line. Size bar = 110 μm. (Middle panels) GABARα2 immunopositive cells are present in hippocampus in CA1, CA3, and dentate gyrus (DG) areas (arrows). Size bar = 100 μm. (Lower panel) DG is shown at higher magnification with GABARα2 immunopositive neurons present (arrow). Note higher intensity of staining in Tg mice compared with C. Size bar = 50 μm.

Previous studies have shown that GABARα2 subunits are expressed widely in the brain, including the hippocampus (Mortensen et al., 2012; Engin et al., 2018). Immunohistochemistry showed that GABARα2 immunoreactivity was increased in the hippocampus, as well as in parietal cortex and in the ventral retrospinal cortex of PGC-1α Tg mice (Figure 2). In the present work, we focused here on the hippocampus, where higher magnifications revealed that GABARα2 is expressed by neurons, as exemplified by immunopositive cells in the dentate gyrus, and in the CA1 and CA3 subregions of the hippocampus (Figure 2B).

### Double Staining of GABARα2 and Gephyrin in the CA1 Region of the Hippocampus

**FIGURE 3:**
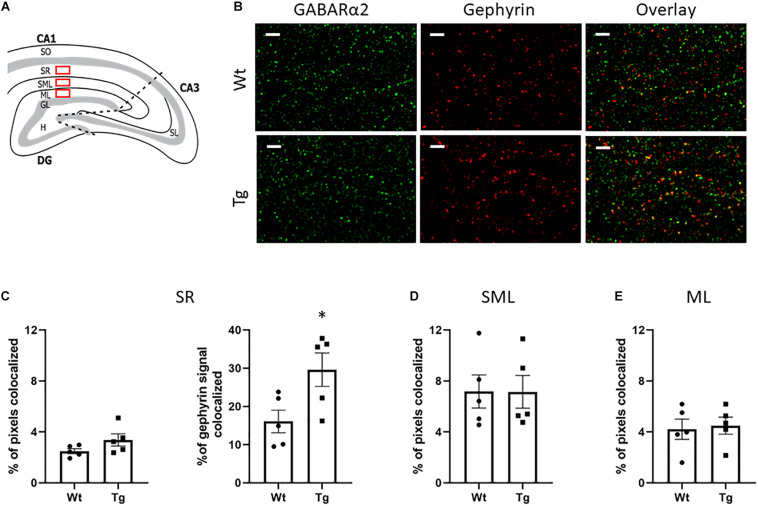
Double immunostaining of GABARα2 with the synaptic protein Gephyrin. **(A)** Schematic picture of the hippocampus showing the CA1, CA3, and DG regions. Double immunostaining was performed in the stratum radiatum (SR), stratum lacunosum moleculare (SLM) layers of CA1 and the adjacent molecular layer (ML) are depicted here as red rectangles. **(B)** Representative confocal images showing immunostaining of GABARα2 (green) and gephyrin (red) in the SR of CA1 region in Wt (upper) and PGC-1α Tg (lower) mice as described in the section “Materials and Methods.” Merged images with colocalization of GABARα2 with gephyrin are in yellow. Size bar = 5 μm. **(C)** Analyses of the SR subregion. Colocalization of GABARa2 and gephyrin was assessed as described in the section “Materials and Methods.” The intensity thresholds for GABARa2 and gephyrin channels were defined using the ImarisColoc tool and the number of colocalized pixes were determined. Quantification was done using unpaired Student’s *t*-test. Values are means ± SEM, *n* = 5 independent animals. In total 2 sections and 4 images per animal were analyzed. (Left) The percentage of colocalized pixels out of total pixel number showed no significant difference between Wt and Tg mice in SR (Wt: 2.493 ± 0.196; Tg: 3.374 ± 0.48; *p* = 0.1). (Right) The percentage of gephyrin signal colocalized with GABARα2 in the gephyrin channel above threshold was significantly increased in the Tg mice (Wt: 16.09 ± 2.973; Tg: 29.62 ± 4.367; *p* = 0.03). **(D,E)** Analyses of SML and ML subregions. There was no difference between Wt and Tg groups with regard to percentage of colocalized pixels out of total numbers in the SLM (Wt: 7.174 ± 1.302; Tg: 7.146 ± 1.285; *p* = 0.9), or in the ML (Wt: 4.212 ± 0.7957; Tg: 4.498 ± 0.671; *p* = 0.8). Quantification was done using unpaired Student’s *t*-test. Values are means ± SEM, *n* = 5.

Gephyrin is a synaptic protein involved in clustering of GABA_A_ receptors in postsynaptic neurons that is important for GABAergic signaling (Tyagarajan and Fritschy, 2014; Choii and Ko, 2015; Pizzarelli et al., 2020). In immunoblots the total level of gephyrin did not change between the groups (data not shown). Pyramidal neurons in the CA1 region take part in the hippocampal circuitry and have apical dendrites in the SR, and SLM (Figure 3A). GABAergic interneurons are also present in these layers. Double immunostaining experiments revealed a co-localization of GABARα2 immunoreactivity with gephyrin in the CA1 region of hippocampus (Figure 3B). Estimation of the percentage of double GABARα2/Gephyrin positive pixels out of the total number of positive pixels showed no obvious change in the SR, in the SLM, or in the molecular layer (ML) between Tg and Wt mice using Students *t*-test (for statistics, see Figure 3C, left and Figures 3D,E). Analyzing the gephyrin channel above threshold showed a significant increase in the percentage of gephyrin signals colocalized with GABARα2 in the SR layer in Tg mice compared with Wt ones using Students *t*-test (Wt: 16.09 ± 2.973; Tg: 29.62 ± 4.367; *p* = 0.03; Figure 3C, right). In contrast, a similar analyses revealed no significant changes between Wt and Tg mice in the ML (Wt: 36,3. ± 6; Tg: 41.1. ± 2; *p* = 0.12), or in the SLM (Wt: 46.6 ± 1,2; Tg: 46.5 ± 1,0; *p* = 0.97) sublayers, suggesting a preferential involvement of neurons in the SR layer in the Tg mice.

### GABAergic Neurotransmission Is Altered in CA1 Hippocampal Region of PGC-1α Transgenic Mice

**FIGURE 4:**
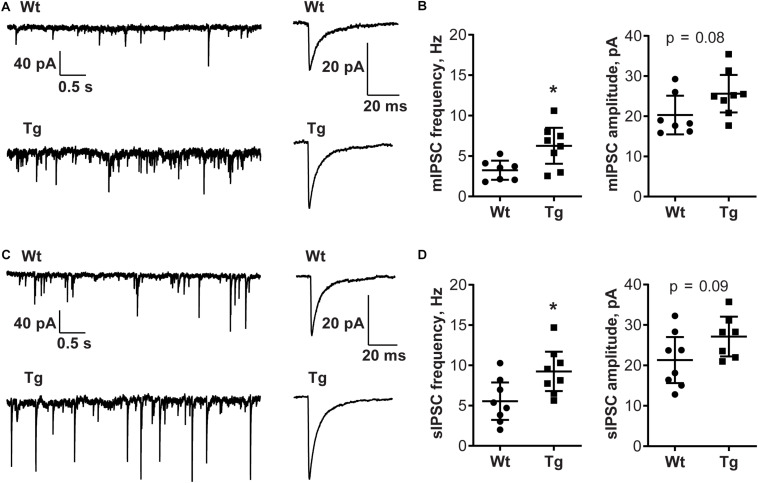
GABA-A transmission is increased in PGC-1α Tg mice. Electrophysiology was done in hippocampus CA1 neurons using parasagittal sections from 4 Wt and 4 PGC-1α Tg mice aged 2 months as described in the section “Materials and Methods.” **(A)** Representative traces of miniature inhibitory postsynaptic currents (mIPSCs) in CA1 pyramidal neurons. Recording and averaged trace of events are shown. **(B)** The frequency of mIPSCs was higher in PGC-1α Tg mice, but not the amplitude, compared with Wt. Quantification was done using unpaired Student’s *t*-test. Values are individual measurements per cell and shown as mean ± SEM, *n* = 7 (Wt), *n* = 8 (Tg). *p < 0.05 for Tg vs. Wt. **(C)** Representative traces of spontaneous inhibitory postsynaptic currents (sIPSCs) in CA1 pyramidal neurons. Recording and averaged trace of events are shown. **(D)** The frequency of sIPSCs was higher in PGC-1α Tg mice, but not the amplitude, compared with Wt. Quantification was done using unpaired Student’s *t*-test. Values are individual measurements per cell and shows as mean ± SEM, *n* = 8 (Wt), *n* = 8 (Tg). *p < 0.05 for Tg vs. Wt.

To investigate whether the alterations in GABARα2 observed in the PGC-1α Tg mice were associated with changes in neurotransmission, we performed patch-clamp recordings in hippocampal slices from control and PGC-1α Tg mice. Data showed that the input resistance and capacitance of cells examined did not differ between Wt and Tg animals (full statistics in Table 2). GABAergic synaptic events were recorded from CA1 pyramidal neurons revealing that the frequency of mIPSCs (Wt: 3.24 ± 0.48 Hz; Tg: 6.27 ± 0.94 Hz, *t* = 2.7, df = 13, *p* = 0.02; Figures 4A,B) and sIPSCs (Wt: 5.55 ± 0.98 Hz; Tg: 9.28 ± 1.03 Hz; *t* = 2.6, df = 14, *p* = 0.02; Figures 4C,D) was higher in the Tg mice compared with Wt. The amplitude of mIPSCs (Wt: 20.31 ± 1.97 pA; Tg: 25.62 ± 1.98 pA; *t* = 1.9, df = 13, *p* = 0.08; Figures 4A,B) and sIPSCs (Wt: 21.31 ± 2.41 pA; Tg: 31.80 ± 4.98 pA; *t* = 1.9, df = 14, *p* = 0.08; Figures 4C,D) followed the same trend, but differences did not reach significance. This demonstrates that the GABAergic neurotransmission is accentuated in the Tg mice, possibly reflecting an increased release probability and enhanced number of GABAergic synapses in the hippocampal neurons. We further noted that the kinetics of the events were the same in the two groups (full statistics in Table 2), which indicates that the channel opening and desensitization of the receptor are not affected in the transgenic mice. Altogether, these results demonstrate that GABAergic neurotransmission is enhanced in the hippocampus of PGC-1α Tg mice compared with Wt ones.

**TABLE 2:** Passive membrane properties and kinetics of mIPSCs and sIPSCs in CA1 pyramidal neurons of control and PGC-1α transgenic mice.

	Wt	PGC-1α Tg	Statistics (unpaired Student’s *t*-test)
Input resistance, MΩ	198.7 ± 39.33 (*n* = 7)	261.9 ± 64.71 (*n* = 8)	*t* = 0.80, df = 13, *p* = 0.44
Capacitance, pF	229.3 ± 37.92 (*n* = 7)	249.0 ± 35.83 (*n* = 8)	*t* = 0.38, df = 13, *p* = 0.71
mIPSC rise (T10/90, ms)	0.40 ± 0.02 (*n* = 7)	0.44 ± 0.03 (*n* = 8)	*t* = 0.97, df = 13, *p* = 0.35
mIPSC decay (τ, ms)	5.0 ± 0.48 (*n* = 7)	5.08 ± 0.44 (*n* = 8)	*t* = 0.15, df = 13, *p* = 0.88
sIPSC rise (T10/90, ms)	0.38 ± 0.03 (*n* = 8)	0.38 ± 0.02 (*n* = 8)	*t* = 0.07, df = 14, *p* = 0.94
sIPSC decay (τ, ms)	4.78 ± 0.31 (*n* = 8)	4.53 ± 0.31 (*n* = 8)	*t* = 0.58, df = 14, *p* = 0.57

### Increased Anxiety-Like Behavior in PGC-1α Transgenic Mice

**FIGURE 5:**
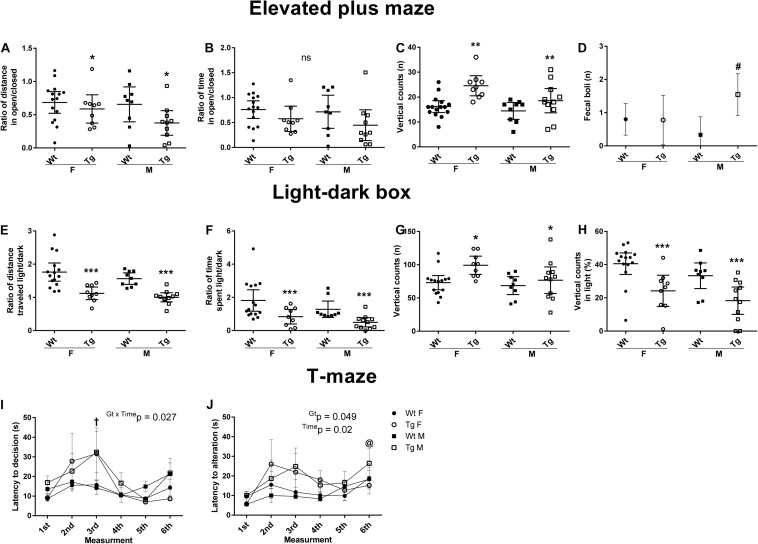
Behavioral changes in PGC-1α Tg mice. 24 wild-types (Wt) and 20 PGC-1α transgenic (Tg) mice were examined using a battery of behavioral tests. Female (F) and male (M) mice were analyzed separately using four different groups: Wt F (*n* = 15), Wt M (*n* = 9), Tg F (*n* = 9) and Tg M (*n* = 11). Data are displayed as mean ± 95% CI [except for panels **(I)** and **(J)** as mean ± SEM]. *p < 0.05, **p < 0.01, ***p < 0.0001 for Tg vs. Wt mice. **(A–D)** Elevated plus-maze (EPM) was carried out to assess anxiety behavior during a 5 min test period. Statistical analyses were performed by using two-way ANOVA, followed by Tukey’s unequal N HSD *post hoc* test. **(A)** The ratio of distance traveled in open vs. closed areas was reduced in the PGC-1α Tg mice. *F*_(__1_, _40__)_ = 4.24, *p* = 0.046, η^2^ = 0.1, power = 0.52. **(B)** The time spent was not significantly different *F*_(__1_, _40__)_ = 3.74, *p* = 0.06, η^2^ = 0.09, power = 0.47. **(C)** The number of vertical counts was higher in Tg mice. *F*_(__1_, _40__)_ = 14.04, *p* = 0.0006, η^2^ = 0.26, power = 0.96. **(D)** The number of fecal boli was increased in PGC-1α Tg male. Gt × Sex *F*_(__1_, _40__)_ = 5.23, *p* = 0.028, η^2^ = 0.12, power = 0.61. **(E-H)** Light–dark box (LDB) test was carried out during a 10 min time period. Statistical analyses were performed by using two-way ANOVA, followed by Tukey’s unequal N HSD *post hoc* test. **(E)** The ratio of total distance traveled. *F*_(__1_, _40__)_ = 32.46, *p* < 0.00001, η^2^ = 0.45, power = 1. **(F)** The ratio of time spent in light vs. dark areas was lower in PGC-1α Tg mice. *F*_(__1_, _40__)_ = 12.26, *p* = 0.0012, η^2^ = 0.45, power = 0.93. **(G)** PGC-1α Tg mice made more vertical counts than Wt. *F*_(__1_, _40__)_ = 8.76, *p* = 0.005, η^2^ = 0.18, power = 0.82. **(H)** The percentage-wise vertical counts made occurred mostly in the dark area. *F*_(__1_, _40__)_ = 19.07 *p* = 0.00009, η^2^ = 0.33, power = 0.99. **(I,J)** T-maze tests were done using six trials during two consecutive days (max 3 min per trial). The mean spatial memory performance during alternation varied between 83 and 86% in all groups. Statistical analyses were performed by using two-way repeated measures ANOVA, followed by Tukey’s unequal N HSD *post hoc* test. **(I)** Latency to decision revealed an overall longer decision time for PGC-1α Tg, especially on the third measurement turn. ^†^*p* < 0.05, Time × Gt interaction, Tg vs. Wt control. **(J)** Latency to alternation showed statistically significant effect. *p* < 0.05, main time effect, respective turn vs. other turns.

Changes in GABA_A_ receptor subunits have been linked to anxiolytic and other types of behavior in experimental animals (Smith et al., 2012; Engin et al., 2018; Skilbeck et al., 2018; Rudolph and Moss, 2019). We were therefore interested to study the behavior of Tg mice with overexpression of PGC-1α in brain neurons. To accomplish this, PGC-1α Tg and Wt mice were subjected to an extensive battery of tests. For this, genotype (Gt) related and sex-specific data are shown separately as indicated in the figures. Full statistics for the animal performance in the different tests are given in the Figure 5, and were analyzed by two-way ANOVA or RM two-way ANOVA, followed by Tukey’s unequal N HSD *post hoc* test.

In the open field test, there were no significant differences between the mice in parameters such as vertical counts, latency to enter the central area; distance traveled in the periphery or the center (data not shown). In the elevated plus-maze (EPM) test, PGC-1α Tg mice showed a preference for closed areas compared with Wt mice, as evident from the reduced ratio of distance traveled in open vs. closed area (Figure 5A). The preference for closed areas of the Tg mice was not significantly different with regard to the time-related ratio (Figure 5B). The number of vertical counts was increased in the transgenic mouse group (Figure 5C). There was also a higher defecation rate among PGC-1α Tg mice (Figure 5D), notably in the Tg male group (*p* = 0.027).

In the light–dark box (LDB) test, Tg mice showed a preference for the dark area, both for the ratio of traveled distance (Figure 5E), and the time spent in light vs. dark (Figure 5F). *Post hoc* analysis revealed lower values for the Tg mice in the distance traveled (*p* = 0.00012), and the ratio of time spent (*p* = 0.0007) in the light area. The total number of vertical counts revealed Gt effect (Figure 5G), as did the ratio of vertical counts in light vs. dark showed (Figure 5H). The Tg mice made more vertical counts (*p* = 0.031), however, these occurred mostly in a dark area (*p* = 0.00016).

Quantifying the total distance there was a difference between sexes with the female in both groups being more active in the EPM (*F*(1, 40) = 9.38, *p* = 0.004), and in the LDB test (*F*(1, 40) = 17.55, *p* = 0.0002). However, the changes in preference for dark area observed between Wt and Tg mice were similar for female and male mice (Figure 5). Together the results show an increased anxiety-like behavior independent of gender in the PGC-1α Tg group compared with Wt.

### PGC-1α Transgenic Mice Exhibit Changes in Learning Behavior

Spontaneous alternation in T-maze is a natural behavior for rodents used to assess cognitive activity (Deacon and Rawlins, 2006). The mean alternation rate of six trials varied between 83 and 86%, indicating good working spatial memory performance in all groups (Figure 5I). However, the Tg mice took a significantly longer time to complete the trials, indicating a possible difference in decision making (Figure 5J). *Post hoc* analysis revealed that the Tg mice were different from Wt on the third turn (*p* < 0.05). Latency to alternation showed a time and genotype effect (*p* < 0.05). Taken together these results indicate that the Wt and Tg mice showed changes in learning ability related to latency measurements in the T-maze test.

### Pioglitazone Elevates GABARα2 Expression in Neurons

**FIGURE 6:**
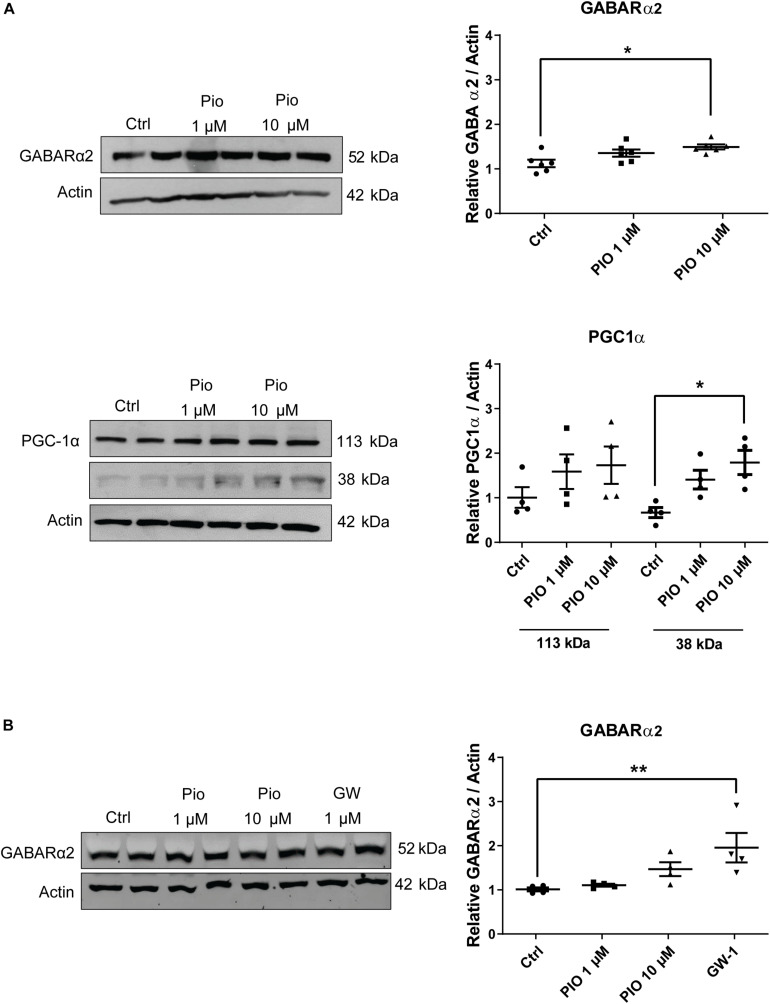
Pioglitazone increases GABA-A receptor α2 and PGC-1α in primary neurons. **(A)** Primary neurons prepared from embryonic E17 rat brain cortex and cultured for 5 days as described in the section “Materials and Methods.” Cells were then stimulated with 1 or 10 μM pioglitazone (PIO) for 24 h followed by immunoblotting using anti-GABARα2 or anti-PGC-1α antibodies and anti-β-actin antibody as control. Quantifications were done by Student’s *t*-test and ANOVA followed by Dunnett *post hoc* test. (Left panel) Immunoblots of GABARα2 (upper lane) and PGC-1α (lower lanes). (Right panel) Quantification using β-actin as control revealed an increase in GABARα2 by 10 μM PIO. Note two isoforms of PGC-1α in the neurons shown as 113 and 38 kDa bands of which the latter was significantly increased by 10 μM PIO. Values are means ± SEM, *n* = 4. *p < 0.05 for 10 μM PIO vs. controls. **(B)** Neurons were stimulated with 1 or 10 μM PIO, or 2 μM PPARγ agonist N-(2-benzoylphenyl)-O-[2-(methyl-2-pyridinylamino) ethyl]-L-tyrosine hydrate (GW-1) for 2 days followed by immunoblotting. Quantifications were done using β-actin as control and Student’s *t*-test and ANOVA followed by Dunnett *post hoc* test. (Left) Immunoblots. (Right) Quantification of GABARα2. Values are means ± SEM, *n* = 4. **p < 0.01 for GW-1 treatment vs. controls. Note, the values for 10 μM PIO in these 2-day treated cultures did not reach statistical significance.

The results above indicate that overexpression of PGC-1α affects GABARα2 subunit expression and behavioral parameters in the PGC-1α Tg mice. PGC-1α acts in conjunction with nuclear transcription factors, such as PPARγ. To investigate whether the activation of PPARγ may influence GABARα2 subunit expression, we employed the drug pioglitazone that acts on PPARγ. Primary cortical neurons were prepared from embryonic rat brain, cultured and stimulated with pioglitazone followed by immunoblotting. Data revealed that 10 μM pioglitazone significantly increased GABARα2 subunit level in the neurons after 1 day (Figure 6A). Pioglitazone further elevated PGC-1α in the neurons, as shown for the 38 kDa neuronal isoform (Martinez-Redondo et al., 2015) (Figure 6A). This indicates a likely positive feedback by which pioglitazone can increase GABARα2 expression in the neurons. Finally, treatment with the PPARγ agonist GW1929 also elevated GABARα2 in the neurons after 2 days (Figure 6B), showing an involvement of PPARγ receptors. Altogether the results show that stimulation of PPARγ can increase GABARα2 subunit expression in primary brain neurons.

## Discussion

PGC-1α plays a physiological role in the regulation of genes contributing to the control of mitochondria functions, cell viability, and general metabolism (see Introduction for references). Impairments in PGC-1α-mediated gene networks are observed in human diseases, including neurodegenerative disorders exemplified by Huntington’s disease (St-Pierre et al., 2006; Soyal et al., 2012; McMeekin et al., 2018) and Parkinson’s disease (Zheng et al., 2010; Clark et al., 2011; Mudò et al., 2012; Jiang et al., 2016; Mäkelä et al., 2016b). As shown previously, PGC-1α can have effects in the brain also on neurotransmission, neuronal connectivity, and behavior (Lucas et al., 2010, 2014; Dougherty et al., 2014; McMeekin et al., 2016, 2020).

PGC-1α is expressed at low levels in the wild-type hippocampus but increased substantially in the PGC-1α Tg mice, as shown previously (Mäkelä et al., 2016a). In the present work, we observed that the transgenic overexpression of PGC-1α in brain neurons increased GABARα2 subunit levels in the hippocampus. This was accompanied by an enhanced GABAergic neurotransmission as studied in the CA1 region of hippocampus, and an increased anxiety-like behavior observed in PGC-1α Tg mice. The role described here for PGC-1α in modulating GABARα2 expression and GABAergic neurotransmission may be important for brain disorders characterized by GABAergic dysfunctions (Lewis et al., 2008; Ramamoorthi and Lin, 2011; McMeekin et al., 2016; Engin et al., 2018).

γ-aminobutyric acid is the major inhibitory neurotransmitter in the brain, and it is present in local interneurons and GABAergic projection neurons (Korpi and Sinkkonen, 2006; Mortensen et al., 2012; Chua and Chebib, 2017). There are two classes of GABA receptors; the ionotropic GABA_A_ receptors which are ligand-gated ion channels, and the metabotropic GABA_B_ receptors which represent G-protein coupled receptors. GABA_A_ receptors are pentameric structures composed of different subunits (Sieghart and Sperk, 2002; Mortensen et al., 2012; Chua and Chebib, 2017). GABA_A_ receptors containing GABARα2 subunits are widespread in different parts of the brain including the hippocampus (Mortensen et al., 2012; Engin et al., 2018). Immunoblotting experiments revealed that GABARα2 subunit levels were increased in hippocampus and in the frontal cortex in the PGC-1α Tg compared with Wt, whilst those of GABARα1 were unchanged. Using anti-GABARα2 antibodies, we noted that there are intensively labeled immunopositive neurons in the CA1, CA3, and DG regions in the hippocampus of TG mice. In addition, cells in the parietal cortex and in the ventral retrosplenial areas were immunostained for GABARα2cin the TG mice, warranting more studies on these brain areas in the future.

Previous studies have shown that endogenous PGC-1α is expressed in the brain by different neurons including interneurons (Cowell et al., 2007; Lucas et al., 2010). In our previous study, we noted an expression of PGC-1α in dopamine neurons in the Thy1-mediated overexpression of PGC-1α in the Tg mice (Mudò et al., 2012). We have previously shown that PGC-1α expression is elevated in the hippocampus in principal neurons in the PGC-1α Tg mice compared with Wt animals (Mäkelä et al., 2016a). Here we further show that cultured cortical neurons express PGC-1α (Figure 6). Of note, recent studies have shown that deletion of PGC-1α in hippocampal pyramidal neurons resulted in neuronal hyperexcitability in the hippocampus (McMeekin et al., 2020). This is in line with our findings here that overexpression of PGC-1α in the PGC-1α Tg mice caused an increase the inhibitory GABAergic drive in the hippocampal neurons. Pyramidal neurons and inhibitory interneurons are functionally coupled in hippocampus indicating that overexpression of PGC-1α in one of the cell types could influence the other one. Preliminary double labeling studies indicated that PGC-1α in the Tg mice is expressed in pyramidal neurons in the hippocampus that are also positive for GABARα2. However, we cannot exclude that some interneurons in the PGC-1α TG hippocampus have also increased PGC-1α expression. Future experiments, employing cell-specific markers and double immunolabeling with PGC-1α *in vivo* and in neuron cultures from Tg animals will be helpful to address this question.

As demonstrated by previous data the number, localization and subunit composition of GABA_A_ receptors are altered in different physiological states, such as during brain development (Yu et al., 2006; Möhler, 2007), following stress (Hsu et al., 2003; Skilbeck et al., 2018), in epilepsy (Raol et al., 2006; Lund et al., 2008), in alcohol dependence (Blednov et al., 2013), in anxiety (Smith et al., 2012; Engin et al., 2016; Rudolph and Moss, 2019), and in neuropsychiatric disorders, including schizophrenia (Lewis et al., 2008; Ramamoorthi and Lin, 2011; Engin et al., 2018).

The GABA_A_ subunits exhibit distinct electrophysiological and pharmacological properties important for their actions in brain circuitry (Sieghart and Sperk, 2002; Korpi and Sinkkonen, 2006; Mortensen et al., 2012; Chua and Chebib, 2017; Engin et al., 2018). In particular, the GABARα2 subunit is considered as the preferential target for the anxiolytic-like effects of benzodiazepine (Smith et al., 2012; Rudolph and Moss, 2019). GABA_A_ receptors containing GABARα2 subunits further contribute to fast network oscillations in the hippocampus that is crucial for memory encoding and retrieval (Heistek et al., 2013), and influence behavioral responses linked to depression and anxiety (Vollenweider et al., 2011; Engin et al., 2016). In view of these findings, there is an increased interest in designing novel drugs specific to GABARα2 subunit for various brain disorders (Engin et al., 2012).

In the present work, we observed that the expression of GABARα2 subunit was increased in the hippocampus of the PGC-1α Tg compared with Wt, whilst that of GABARα1 were unchanged. To study the physiology of this, we performed electrophysiology experiments in the CA1 region revealing that the frequency, but not the amplitude of sIPSCs and mIPSCs was enhanced in the hippocampus in Tg compared with Wt mice. Changes in the frequency of spontaneous and miniature synaptic currents may be caused both by alterations in release probability (via presynaptic mechanism), and by changes in the number of functional synapses (Glasgow et al., 2019). To investigate this further we examined the extent to which the GABARα2 subunit is associated with gephyrin in postsynaptic clusters in the CA1 region of hippocampus. Gephyrin is a large scaffolding protein in the postsynaptic compartment of inhibitory neurons (Tyagarajan and Fritschy, 2014; Choii and Ko, 2015). Reduced GABA_A_ receptors clustering is known to enhance anxiety-like behavior (Crestani et al., 1999). Using an assay for colocalization of pixels developed earlier (Costes et al., 2004), we analyzed the gephyrin channel above threshold. Results showed that there is an increase in the number of GABARα2 pixels in the SR layer in the TG compared with Wt animals. This indicates a higher number of GABARα2 subunit in association with postsynaptic gephyrin structures in the CA1 region of the hippocampus in PGC-1α Tg mice compared with Wt. Interestingly, in the ML and SML sublayers of CA1 no such increases in the percentage of gephyrin signals colocalized with GABARα2 were noted. This may suggest that the dendritic interneurons, which innervate pyramidal cells in SR, are particularly affected in the Tg mice. These may include, among others, parvalbumin-positive bistratified cells, cholecystokinin-positive Shaffer collateral-associated cells and somatostatin-positive neurogliaform cells (Booker and Vida, 2018). The precise modulations of the hippocampal circuitry in the PGC-1α Tg warrant further studies.

Our observations showing an increase in the expression of GABARα2 and its co-localization with gephyrin favor the possibility that the elevated frequency of mIPSCs is related to the higher number of active GABAergic synapses on CA1 pyramidal cells. However, we cannot exclude a function of PGC-1α in enhancing the release probability in GABAergic synapses in the CA1 region. It is possible that overexpression of PGC-1α alters the excitation/inhibition balance in hippocampus as well as in other brain regions by increasing the number of inhibitory synapses on principal neurons. with behavioral consequences and will require further studies. In particular in the hippocampus, the roles of ventral and dorsal hippocampus separately would be important to study in more details.

To measure possible changes in behavior in the Tg mice, we focused on the hippocampus, which is an important area for learning, memory processing, stress responses and anxiety-like behavior (Engin et al., 2016; Padilla-Coreano et al., 2016: Jimenez et al., 2018). Results obtained with the EPM and LDB tests indicated that the PGC-1α Tg showed an increase in anxiety-like behavior. Thus, the Tg mice were found to spend less time in the open area compared with the Wt mice and showed a higher number of vertical counts in the dark. These findings demonstrate an enhanced anxiety-like behavior in the PGC-1α Tg mice compared with Wt.

Previously, it has been shown that intrahippocampal infusion of GABA-A receptor agonist inhibit anxiety reactions (Engin and Treit, 2007). This is seemingly at variance with present findings of an increased anxiety-like behavior in the PGC-1α Tg mice. As shown by Zhang et al. (2014), ventral and dorsal hippocampus play contrasting roles in anxiety regulation in mice. Thus, anxiety-related behavior is regulated by ventral hippocampus-amygdala-medium prefrontal cortex circuitry (Adhikari, 2014). Specific lesions or inhibition of ventral hippocampus impair acquisition of conditioned freezing in fear conditioning paradigm (Maren and Holt, 2004), and severely affect behavior related to unconditioned fear (e.g., entrance to open arms in an elevated plus maze test (Kjelstrup et al., 2002). Anxiety evokes an increase in the synchrony of mPFC and ventral hippocampus EEG in the theta range, and inhibition of hippocampal input to the prefrontal cortex produced an anxiolytic effect (Padilla-Coreano et al., 2016). Stimulation of inputs from basolateral amygdala to the CA1 area of ventral hippocampus evokes anxiety-like behavior (Pi et al., 2020). Increased inhibition of the CA1 pyramidal cells in the PGC-1α Tg animal model may disturb the function of mPFC-vHIPP-amygdala circuit, causing anxiety-like behavior.

In addition, the contribution of other brain areas to the observed behavioral phenotype cannot be excluded. In this work, we have shown increased inhibition in the CA1 region and anxiety phenotype in the mouse behavior. As such these data do not establish a direct causal relationship between the altered inhibition in hippocampal circuitry caused by PGC-1α overexpression and the observed anxiety-like behavior. Additional experiments including rescue of initial PGC-1α levels are needed to demonstrate causality links between gene expression and behavior. In particular, the specific roles of ventral and dorsal hippocampus in the anxiety-linked behavior in PGC-1α Tg mice would be important to study in more detail in the future.

To investigate spatial learning in the Tg mice, we employed the T-maze test. In this test, Wt and Tg mice behaved rather similarly, however, the time to reach a decision in the T-maze was relatively longer in the Tg mice. This may indicate difficulties in decision-making, possibly related to increased anxiety. To decipher the behavior of Tg mice compared with Wt in more detail, tests such as Barnes maze and other learning tests could be valuable to perform. There is also drugs available acting on specific GABA_A_ receptors that could be helpful in this context. For example, clinical studies employing the GABARα2 specific compound, MK-077, have revealed an improvement in cognition and gamma-band oscillations in the frontal cortex of schizophrenia patients (Lewis et al., 2008). Whether such alterations are also found in brains of PGC-1α Tg mice will require further investigations.

In the brain, there is a complex regulation of the different GABA receptors by various agents and factors (Korpi and Sinkkonen, 2006). Brain-derived neurotrophic factor (BDNF) was shown to increase the expression of GABARα4 subunits in the neurons via stimulation of the early response factor 3 (Roberts et al., 2006). BDNF, however, reduced GABARα1 subunit expression through a Janus kinase/signal transducer and activator of transcription pathway (Lund et al., 2008). BDNF was reported also to induce PGC-1α in cultured hippocampal neurons (Cheng et al., 2012). In the present work, we noted that the expression of GABARα2 subunit was increased in the Tg mice related to enhanced levels of PGC-1α in brain neurons. This was corroborated in experiments using the drug pioglitazone and the agonist, GW1929 to stimulate PPARγ in primary hippocampal neurons. Thus, pioglitazone and GW1929 both increased the mRNA levels of GABARα2 subunits in the neurons within 1–2 days.

The precise mechanisms for the upregulation of GABARα2 in the neurons warrant further investigations including the use of gene promoter constructs. In our study, we have focused on PPARγ but it is clear that PGC-1α as a transcriptional coactivator can interact with other transcription factors as well and this may vary between tissues. We are currently studying which of several transcription factors PGC-1α preferentially interacts with in brains of control and TG mice. Furthermore, it is likely that other genes than GABARα2 are altered in the PGC-1α Tg mice and may contribute to the observed changes in GABAergic transmission and behavior as studied in the present paper. In future work, we will investigate these gene products in detail, including calcium-dependent proteins involved in neurotransmitter release in the brain.

From the clinical point of view, thiazolidinedione drugs, like pioglitazone, are used in the treatment of metabolic diseases such as type-2 diabetes. An increasing amount of data suggests that such drugs and compounds acting on PPARγ-PGC-1α signaling may have beneficial effects in different brain diseases (Patrone et al., 2014; Carta and Simuni, 2015; Mäkelä et al., 2016b). Adding to this we show here that pioglitazone can increase expression of GABARα2 subunits in brain neurons with possible functional consequences for inhibitory neurotransmission that will require further investigations. In conclusion, the role described here for PGC-1α in increasing GABARα2 subunit expression and GABAergic neurotransmission may be important in different neuropsychiatric disorders and other brain diseases.

## Data Availability Statement

The raw data supporting the conclusions of this article will be made available by the authors, without undue reservation.

## Ethics Statement

The animal study was reviewed and approved by the Ethics Committee and the Koe-eläinkeskus University of Helsinki.

## Author Contributions

LK and DL designed the project. TV, SM, DP, AS, CP, EV, TTi, OE, ML, TTa, LK, VV, and DL designed the experiments. TV, SM, DP, AS, CP, VS, JN, MU, MK, RM, TTi, and VV performed the experiments. TV, SM, AS, ML, VV, and DL wrote the manuscript. All authors analyzed the experiments, edited and approved the manuscript.

## Conflict of Interest

TV and JN were employed by Quretec Ltd., Tartu, Estonia and TT and MU by Protobios LCC, Tallinn. The remaining authors declare that the research was conducted in the absence of any commercial or financial relationships that could be construed as a potential conflict of interest.

## Abbreviations

BDNF: brain-derived neurotrophic factor
EPM: elevated plus-maze
GABA: γ-aminobutyric acid
GABA_A_ receptor: inotropic GABA receptor
GABA_B_: metabotropic GABA receptor
GABAR α 1: γ-aminobutyric acid (GABA) type A receptor- α 1 subunit
GABAR α 2: γ-aminobutyric acid (GABA) type A receptor- α 2 subunit
mIPSCs: miniature inhibitory postsynaptic currents
PGC-1α: Peroxisome proliferator-activated receptor- γ coactivator-1 α
PPARγ: peroxisome proliferator-activated receptor γ
sIPSCs: spontaneous inhibitory postsynaptic currents
Tg: transgenic mice
Wt: wild-type mice.
